# Improvement of quality of life in diabetic patients treated with percutaneous transluminal angioplasty

**DOI:** 10.1097/MD.0000000000012228

**Published:** 2018-10-12

**Authors:** Fernando Luis Bernal Páez, Miguel Alcaraz Baños, Jose Manuel Felices Abad, Ana Bernal Belmonte, Gabriel Gijon-Nogueron, Manuel Pardo Rios

**Affiliations:** a Nursing Department, Faculty of Nursing; b Head of Radiology and Physical Medicine Department. Faculty of Medicine and Nursing; c Radiology and Physical Medicine Department; d, Nursing Department. Faculty of Nursing. Faculty of Nursing.; e Department of Nursing and Podiatry, University of Malaga. IBIMA. Spain; f Catholic University of Murcia (UCAM), Murcia. Spain.

**Keywords:** critical limb ischemia, diabetes mellitus, transcutaneous oxygen tension and ankle brachial index

## Abstract

To assess the quality of life (QoL) of treated patients in order to evaluate the success of peripheral transluminal angioplasty (PTA) and correlate physical parameters with clinical progress at 6 months post-PTA.

According to TASC II classifications, 69 patients were selected for PTA. Clinical evaluation and diagnostic tests were performed before, after and after 6 months following PTA. The SF-36 QoL questionnaire was added as an additional parameter.

Fifty one patients were included in the study. The ankle-brachial index (ABI) increased from 0.49 ± 0.11 before PTA, to 0.81 ± 0.14 after PTA (*P < *.001) and 0.76 ± 0.10 at 6 months following PTA (*P < *.001). Transcutaneous oxygen pressure (TcPO_2_) increased from 28.05 ± 3.15 mm Hg before PTA, to 39.89 ± 4.12 after PTA (*P < *.001) and 46.4 ± 3.81 at 6 months following PTA (*P < *.001). The lumen of the affected blood vessel increased from 29 ± 18% before PTA, to 81 ± 10.3% after PTA (*P < *.001). SF-36 values increased from 29 ± 18 before PTA, to 81 ± 10.36 at 6 months following PTA (*P < *.001).

The improvement of QoL is the parameter that best describes the symptoms and functionality of the patient, therefore, should be used to determine the successful PTA. Although ABI and TcPO_2_ with arteries functionality and tissue oxygenation, they are not show a significant correlation with all parameters determined in the QoL questionnaire.

## 1 Introduction

Intermittent claudication (IC) is the clinical diagnosis given for muscle pain, classically the calf muscle, which occurs during exercise and is relieved by a short period of rest. IC is one of the most common clinical manifestations of critical limb ischemia (CLI) and is therefore very common in long-term diabetic patients. CLI is defined as the presence of chronic ischemic pain at rest, ulcer or gangrene in lower extremities attributable to objectively proven peripheral arterial occlusive disease.^[1,2]^ Percutaneous transluminal angioplasty (PTA) has increased in use as treatment in diabetic patients with peripheral arterial occlusive disease.^[2,3]^

The evaluation of patients with IC is often based on skin assessment, distance until intermittent claudication, ankle-brachial index (ABI), and arteriography. In recent years, transcutaneous oxygen pressure (TcPO_2_) has proven a solution and alternative to assessing PTA results.^[4–6]^

The hypothesis is that the parameters analyzed (ABI, TcPO_2_, and degree of stenosis by arteriography) neither report on the clinical course of the patient, improved QoL, nor do they establish a prognosis. In the study hereby presented, we assessed the QoL of treated patients in order to evaluate the success of PTA together with the aim of correlating physical parameters (ABI and TcPO_2_) with patients’ clinical progress 6 months following PTA, thus determining which prognostic factor is responsible for the success of PTA.

## 2 Material and methods

### 2.1 Patient selection

From 1 July 2013 to 31 July 2017, an observational study was carried out with a total of 95 consecutive ischemic limb patients were admitted to the Vascular Radiology Service of our Hospital, for clinical evaluation and diagnostic tests (IC, ABI, TcpO_2_ and Arteriography or CT angiography). The investigation was approved by Ethical Committee of the Institution and conforms to the principles in the Declaration of Helsinki. Patients signed consent to participation in this study. Duplex scanning was performed in cases with reduced or absent foot pulses, ABI < 0.90 (in the absence of arterial calcification), or TcPO_2_ < 50 mm Hg (in the absence of edema). If at least 2 of these 4 examinations were abnormal, arteriography was carried out and PTA revascularization performed concomitantly. Of the original 69 patients, 51 (15 women and 36 men) with a mean of age of 73.7 ± 7.6 years (between 48 and 86 years) were selected by the hospital's protocol for PTA, according to *Trans-Atlantic Inter-Society Consensus* II (TASC II) criteria. In patients for whom PTA was attempted unsuccessfully, a by-pass operation was considered, depending on the severity of the obstructive disease and surgical risk factors. All patients were scheduled for follow-up controls at 6 months following completion of the PTA. A minimal rate of involuntary attrition (death, difficulty transporting to hospital or other factors) meant that 51 patients comprised the study population.

### 2.2 TcPO_2_ measurement

Values for TcPO_2_ were determined for all patients included in the study before PTA. Measurements were repeated after PTA and at 6 months. According to established recommendations,^[1]^ TcPO_2_ measurements were taken at the dorsum of the foot with the patient resting in the supine position in an air-conditioned room maintained at 22°C and the electrode at 44°C. The instrument used was a TCM3 (Radiometer, Copenhagen, Denmark) equipped with a Clark electrode.

### 2.3 ABI measurement

All patients included in the study had ABI measurements taken before PTA, after PTA and at 6 months. According to standard procedure, ABI was measured using a sphygmomanometer cuff placed just above the ankle, and a Doppler instrument with an 8 MHz frequency (Multi-Dopplex II, Huntleigh Healthcare Limited, Cardiff, UK).

### 2.4 PTA procedure or interventional technique

PTA was indicated when angiographically or CT angiography reported obstructions of greater than 50% of the vessel lumen. The PTA revascularization technique was employed extensively: no stenosis or occlusion was considered unsuitable, *a priori,* for PTA recanalization. Stenosis and occlusion >10 cm in length, consecutive multiple stenosis, calcified occlusions, were all treated by PTA. Vessel recanalization was considered complete when direct flow was obtained, with no significant residual stenosis along the artery.

PTA was performed under local anesthesia through anterograde puncture of the ipsilateral common femoral artery. If obstructive atheroma was present on duplex scanning of the iliac trunk or common femoral artery, the puncture was performed by means of a contralateral approach. A 6-French vascular introducer was positioned to perform a preliminary angiographic study using diluted (50%) nonionic contrast medium. An angiographic or PTA 0.014 to 0.035 in. guide wire was inserted to pass through any arterial obstructions and a 3.0 to 5.0 French balloon catheter, was used to dilate 2 to 8 mm diameter arteries. Vessel recanalization was considered successful if direct flow was obtained in the treated vessel with no residual stenosis along > 30% of the vessel diameter over the entire artery. Stents were placed if they were deemed necessary by the interventional radiologist, mainly in the presence of severe dissection, parietal thrombus, >30% remaining stenosis, or recoil.

### 2.5 Quality of life questionnaire

The SF-36 health assessment survey offers patients the opportunity to make an informal estimate of their own overall health and well being. The survey measures 8 areas of health: physical functioning, physical role functioning, bodily pain, general health perceptions, vitality, social role functioning, emotional role functioning, and mental health. The questionnaire was administered before PTA and repeated at 6 months following PTA. Patients were instructed to indicate the most appropriate response in their opinion. The SF-36 questionnaires were scored, and the scores transformed as recommended in the SF-36 scoring guidelines, with higher scores indicating a better QoL. For each subscale item, scores were recorded, summed, and standardized into a scale from 0 to 100, with better health states resulting in higher scores.

### 2.6 Statistical methods

The statistical analysis consisted of comparing pre and post-treatment results by equality of means for paired data with a paired Student's *t*-test. The relationships between variables were established by regression/correlation analysis using Pearson's linear correlation coefficient. The results were considered significant when *P* was <.05 (*P < *.05). Descriptive data are given as means ± standard deviation.

## 3 Results

### 3.1 Patient population

In 80 patients, at least one pedal pulse was absent, TcPO_2_ was < 50 mm Hg and duplex scanning showed evidence of significant stenosis. According to TASC II classifications, lesions were: 24 Type I, 39 Type II, 11 Type III and 6 Type IV. PTA was successfully performed during the same session as arteriography in 69 patients. Of the 63 patients with lesions Type I and II who began the study, only 51 patients remained as participants at the 6 month stage and therefore only their data are presented.

### 3.2 PTA technical effectiveness

The lumen of the artery increased from 29 ± 18% before PTA to 81 ± 10.3% following PTA (*P < *.001). ABI increased from 0.49 ± 0.11 before PTA, to 0.81 ± 0.14 following PTA (*P < *.001) and 0.76 ± 0.10 at 6 months following PTA (*P < *.001). ABI was erroneous or could not be used (false negatives and false positives) for 13 of the patients studied; in 8 of these cases, it was impossible to determine ABI since there was no signal or the artery could not be compressed, while in 5 cases a false negative was obtained (i.e. ABI was normal yet high levels of arterial stenosis and/or a low TcPO_2_ were present). TcPO_2_ was measured in all treated patients and showed an increase from 28.05 ± 3.15 mm Hg before PTA, to 39.89 ± 4.12 following PTA (*P < *.001); 6 months following PTA, the value for TcPO_2_ reached 46.4 ± 3.81 (*P < *.001).

### 3.3 Quality-of-life questionnaire (SF-36)

**Figure 1:**
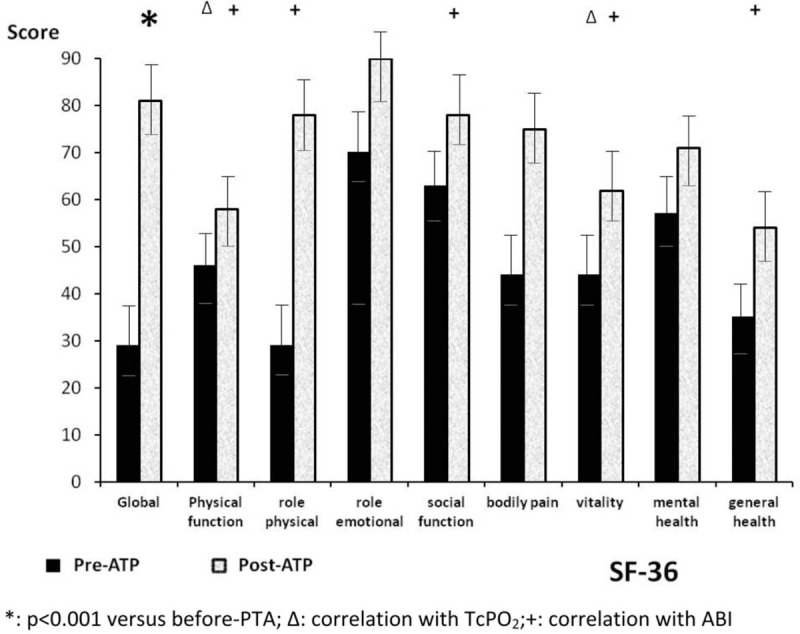
Score for quality of life questionnaire SF-36 including each of the categories.

QoL scores via SF-36 increased from 29 ± 18 before PTA to 81 ± 10.36 six months following PTA (*P < *.001). Figure 1 shows the increase in scores for the QoL questionnaire both before and after PTA in each category of the SF-36 and in Table 1 the modified scores for the questionnaire, both before and after PTA, are shown in comparison with healthy adults.^[12]^

**Table 1:**
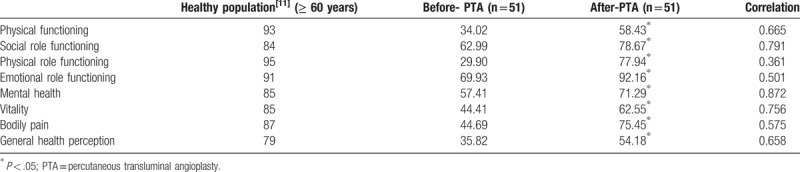
Modification of scores between categories of the quality of life questionnaire SF-36 before and after PTA.

### 3.4 Correlation between variables

Table 2 shows the statistical analysis and correlations between ABI and TcPO_2_ with each of the categories of the SF-36 QoL questionnaire. The correlation between TcPO_2_ and ABI is very low (*r* = 0.22; *P < *.174). However there is a correlation between the score obtained by the SF-36 questionnaire and the physical parameters studied (Table 2). Thus TcPO_2_ is correlated with physical function (*P < *.001) and vitality (*P < *.05); while ABI is correlated with the perception of general health (*P < *.001), as well as with vitality (*P < *.001) and physical function, social function and physical role (*P < *.05).

**Table 2:**
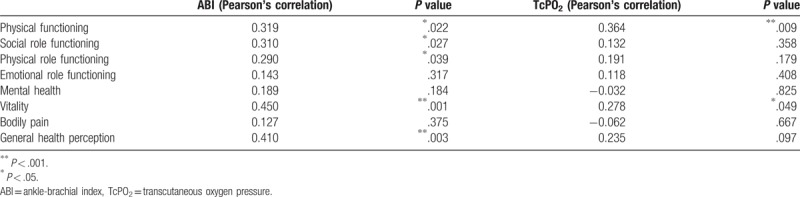
Correlation between categories of quality of life questionnaire SF-36, ABI, and TcPO_2_.

## 4 Discussion

The usefulness of TcPO_2_ in assessing the effectiveness of PTA was found,^[4]^ in patients where it was not possible to determine ABI,^[5]^ and even as a prognostic factor with greater specificity, since the increase of TcPO_2_ continues until 6 months after the PTA.^[6]^ Furthermore, our results reveal one limitation of ABI is the existence of “false negatives” in the presence of partial arterial stenosis with calcified walls, thus portraying a normal ABI patient in the presence of CLI. Another clinically relevant phenomenon is that distal embolization can be induced following PTA treatment of stenosis^[7,8]^ due to residual particles of the atheromatous plaque.

This might explain the reduction of O_2_ in some patients in whom an increase in the arterial diameter is not followed by a concomitant increase in TcPO_2_ but, paradoxically, a decrease. Also some results of patients indicate that lesions or sores that do not improve despite the success of revascularization after PTA. For these reasons, in the last years the importance of combining imaging techniques with physiological tests to assess the results of the ATP is emphasized.^[9]^

The parameters previously studied (ABI, TcPO_2_, or arteriography) do not reveal the clinical course of the patients, neither improved QoL, nor establish a long-term prognosis. Comparison of the physical parameters together should be focused to clinic success achieved.^[2]^ Our results show that PTA produces a significant increase in scoring the QoL of patients showing clinical improvement; at least during the first 6 months and that they achieve a significant increase also in the 8 specific categories which evaluates the QoL questionnaire SF-36.

In a review of instruments of QoL related to health in patients with ischemic heart disease, Demspter and Donnelly^[10]^ conclude that the SF-36 is the best instrument by psychometric evidence presented. The QoL scores obtained in our patients does not show a correlation with the physical parameters that determine the success of the PTA. Only partial aspects of QoL questionnaire (physical function, role-physical, bodily pain, general health, vitality, social function, role-emotional, mental health) reveal a statistically significant correlation. TcPO_2_ was found to correlate with physical function and vitality; while ABI reached statistical significant levels with the social function, vitality and general health perception.

These seemingly contradictory results could be explained by 2 different circumstances: on the one hand, the difficulties of quantifying the health status through a questionnaire specific for diabetic and elderly patients with multiple pathologies that may require more number of patients to achieve statistical significance; on the other hand, it could also be explained by the statistical bias of ABI since patients with severe stenosis are not assessed.

With this work we have been able to show the difficulty of the assessment of patients with arterial disease. Historically assessments have focused on the macro and micro angiopathy but we consider essential to consider increasing functionality and QoL of patients who are treated by PTA. The problem is to select the parameter that is most representative of health and patient improvement: our view is like that of other authors say that the main indicator is the QoL complemented with other the tests.^[11]^ A limitation of this study is that none of the 3 parameters analyzed provides a clinical prediction of limb salvage. Future studies should be studied to better indicator to determine the limb salvage and de QoL.

Currently there are a lot of diagnostic tests that in addition to providing static images, allows professionals to obtain videos (i.e., cine MRI). The interpretation of this type of evidence is complex since it implies showing attention to different areas that are constantly changing. New technologies, such as the computational models published by Kong et al,^[12]^ are facilitating the interpretation of medical images for the diagnosis of vascular functionality. Computational fluid dynamics is an increasingly used method for investigation of hemodynamic parameters and their alterations under pathological conditions, which are important indicators for diagnosis of cardiovascular disease.^[13]^ Some authors have proposed models to assess the motion of the common carotid artery wall, who has been established to be useful in early diagnosis of atherosclerotic disease.^[14]^ In the following years, this type of models and computational analysis will be extended to other vascular areas such as peripheral arterial disease, helping to improve the accuracy and effectiveness of the diagnoses.

The improvement of the QoL is the parameter that best describes the symptoms and functionality of the patient, therefore, should be used to determine the successful PTA. Although ABI is associated with arterial functionality and TcPO_2_ with tissue oxygenation, they are not show a significant correlation with all parameters determined in the QoL questionnaire.

## Author contributions

**Conceptualization:** Fernando Luis Bernal Páez, Miguel Alcaraz Baños, Jose Manuel Felices Abad, Gabriel Gijon-Nogueron, Manuel Pardo Ríos.

**Data curation:** Ana Bernal Belmonte.

**Formal analysis:** Miguel Alcaraz Baños, Manuel Pardo Ríos.

**Investigation:** Fernando Luis Bernal Páez, Miguel Alcaraz Baños, Jose Manuel Felices Abad, Ana Bernal Belmonte, Gabriel Gijon-Nogueron, Manuel Pardo Ríos.

**Methodology:** Miguel Alcaraz Baños, Gabriel Gijon-Nogueron, Manuel Pardo Ríos.

**Supervision:** Miguel Alcaraz Baños, Jose Manuel Felices Abad.

**Validation:** Manuel Pardo Ríos.

**Writing – original draft:** Fernando Luis Bernal Páez, Miguel Alcaraz Baños, Gabriel Gijon-Nogueron, Manuel Pardo Ríos.

Manuel Pardo Ríos orcid: 0000-0001-7965-0134
